# A spatiotemporal land-use-regression model to assess individual level long-term exposure to ambient fine particulate matters

**DOI:** 10.1016/j.mex.2019.09.009

**Published:** 2019-09-12

**Authors:** Tao Liu, Jianpeng Xiao, Weilin Zeng, Jianxiong Hu, Xin Liu, Moran Dong, Jiaqi Wang, Donghua Wan, Wenjun Ma

**Affiliations:** a Guangdong Provincial Institute of Public Health, Guangdong Provincial Center for Disease Control and Prevention, Guangzhou, 511430, China; b General Practice Center, Nanhai Hospital, Southern Medical University, Foshan, 528200, China

**Keywords:** Spatiotemporal land-use-regression (ST-LUR) model, Air pollution, Exposure assessment, Land use regression model, Human health

## Abstract

We aimed to establish a spatiotemporal land-use-regression (ST-LUR) model assessing individual level long-term exposure to fine particulate matters (PM_2.5_) among 6627 adults enrolled in Guangdong province, China from 2015 to 2016. We collected weekly average PM_2.5_ concentration (from the air quality monitoring stations) and visibility, population density, road density and types of land use of each air quality monitoring station and participant’s residential address from April 2013 to December 2016. A ST-LUR model was established using these spatiotemporal data, and was employed to estimate the weekly average PM_2.5_ concentration of each individual residential address. Data analysis was carried out by R software (version 3.5.1) and the *SpatioTemporal* package was used. The results showed that the ST-LUR model applying the land use data extracted using a buffer radius of 1300 m had the best modelling fitness. The results of 10-fold cross validation showed that the R^2^ was 88.86% and the RMSE (Root mean square error) was 5.65 μg/m^3^. The two-year average of PM_2.5_ prior to the date of investigation were calculated for each participant. This study provided a novel method to precisely assess individual level long-term exposure to ambient PM_2.5_, which may extend our understanding on the health impacts of air pollution.

• Variables input in the spatiotemporal land-use-regression (ST-LUR) model include visibility, population density, road density, and types of land use.
• The land use data should be extracted using a buffer radius of 1300 m.
• The R^2^ of the ST-LUR model was 88.86% and the RMSE was 5.65 μg/m^3^, indicating the good performance of the model.

## Graphical abstract

**Figure:**
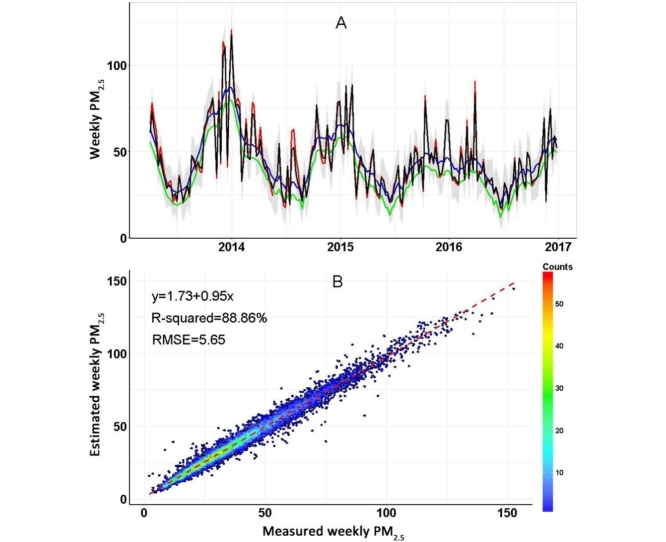

**Specification Table**

**Table:** 

Subject Area:	Environmental Science
More specific subject area:	Guangdong province, south China
Method name:	Spatiotemporal land-use-regression (ST-LUR) model
Name and reference of original method:	https://cran.r-project.org/web/packages/SpatioTemporal/index.html
Resource availability:	Data

## Method details

This is a community based cross-sectional study conducted in Guangdong province located in south China. We aimed to estimate individual participant’s long-term exposure to ambient fine particulate matters (PM2.5), and further assess the effects of PM_2.5_ exposure on risks of chronic diseases. Participants aged over 18 years old were selected from 14 districts or counties using a multistage cluster sampling method which has been described elsewhere [1,2]. Residential address of each participant was obtained by a questionnaire interview.

**Fig. 1:**
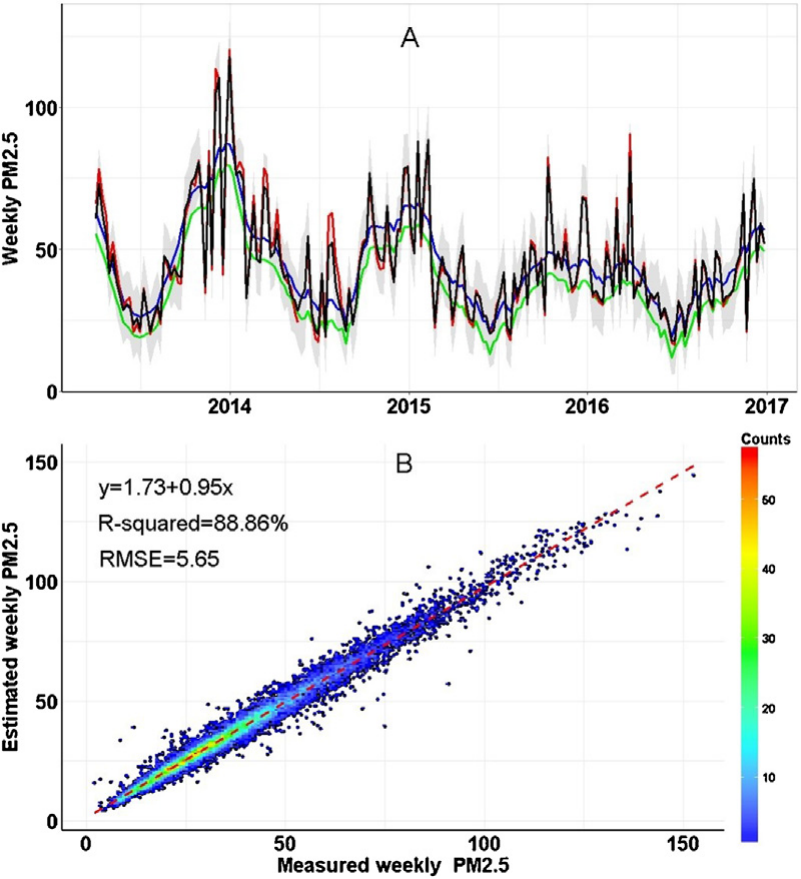
Performance and validation of modeling predicting the weekly PM2.5. Panel A: The predicted and observed data for one monitoring station. The red line denotes observed weekly PM_2.5_ concentration. The black line and grey shading give predictions and 95% CIs. The green and blue lines give the contributions of temporal trends (temporal basis functions). Panel B: Density scatterplots of model performance. RMSE, root mean squared prediction error (μg/m^3^).

**Fig. 2:**
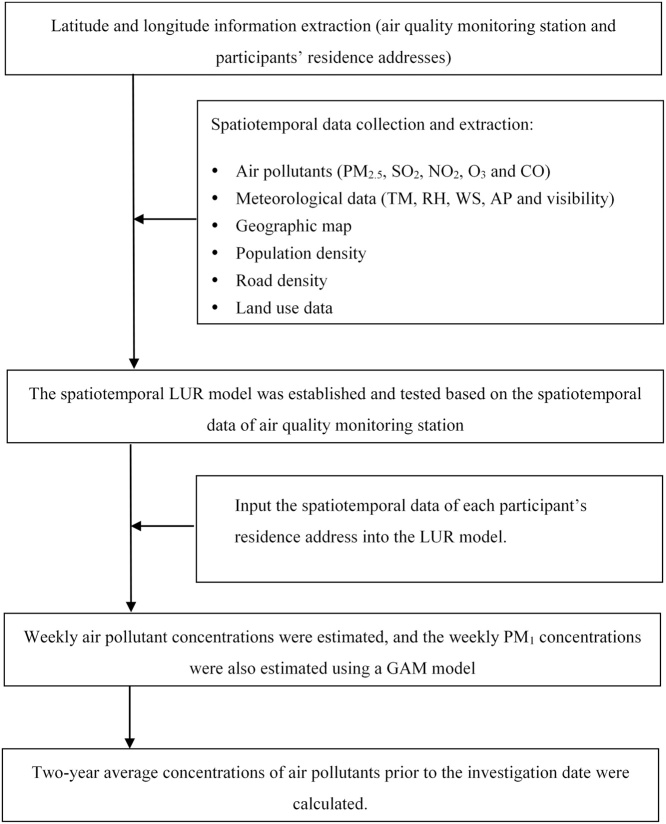
The process of long-term exposure assessment to air pollutants.

A spatiotemporal land use regression (ST-LUR) model was used to assess the individual level two-year average exposure to ambient air pollutants including PM_2.5_. First, we collected daily ambient air pollutant data (April 2013 to December 2016) of 55 air quality monitoring stations which began to monitor PM_2.5_ in April 2013 in Guangdong province from the National Urban Air QualityReal-time Publishing Platform (http://106.37.208.233:20035/), meteorological data [daily mean temperature (TM), relative humidity (RH), mean wind speed (WS), atmospheric pressure (AP), and visibility] of all 86 monitoring stations from Guangdong Meteorological Service, population density data in 2015 from GeoData Institute in University of Southampton (www.worldpop.org.uk), and geographic information system (GIS) covariates (geographic map, road density, and land use data) from the Data Center for Resources and Environmental Sciences (http://www.resdc.cn). The detailed information of these data sources is shown in supplementary material (Table 1). Second, we prepared the data for the ST-LUR model establishment. We extracted the latitude and longitude information of each residence address and air quality monitoring station. The meteorological data of each air quality monitoring station and participant’s residence address was extracted using an Inverse Distance Weighted method with a resolution of 1*1 km. Similarly, we extracted the population density, length of road, and types of land use data (farmland, blue space, living land, and green space) of each address (both monitoring station and residence address) within different buffer radiuses (300 m, 500 m, 800 m, 1000 m, 1300 m, 1500 m, 1800 m, and 2000 m). Then we calculated the weekly averages of PM_2.5_ concentrations and meteorological data for each address. Third, we established a ST-LUR model using the above prepared data of all air quality monitoring stations: weekly air pollutant, weekly visibility, population density, road length and land use data. Two smooth temporal basis functions were included in the model. In particular, we selected the visibility as an important covariate because it is a good predictor of air quality [3]. The process of model establishment has been well demonstrated in the *SpatioTemporal* R package [4]. The results of modelling showed that extraction of land use data within a radius of 1300 m could produce the best modelling fitness (Table 2). The 10-fold cross validation analyses showed that the R^2^ was 88.86% and RMSE (Root mean square error) was 5.65 μg/m^3^ (Fig. 1). Fourth, we input the predictors of each participant’s address into the model, and predicted the weekly air pollutant concentrations from April 2013 to December 2016. Finally, the two-year average of air pollutant concentrations prior to the date of investigation were calculated for each participant. The entire process of air pollution exposure assessment can be seen in Fig. 2.

**Table 1:** Variables used in the spatiotemporal land use regression (ST-LUR) model.

Variable	Brief description	Time	Format of data	Source
Air pollutants	PM_2.5_, SO_2_, NO_2_, O_3_ and CO	2013–2016	Weekly mean concentration	National Urban Air Quality Real-time Publishing Platform
Meteorological data	TM, RH, WS, AP and Visibility in each address were extracted with a resolution of 1.0*1.0 km	2013–2016	Weekly mean	Guangdong Meteorological Service
Population density	Population density	2015	Raster data with a resolution of 0.1*0.1 km	GeoData Institute
Road density	Distribution of road	2016	Vector data	Data Center for Resources and Environmental Sciences
Types of land use	The types of land used were divided into four groups: farmland, blue space, living land, and green space.	2015	Raster data with a resolution of 0.03*0.03 km

**Table 2:** Comparisons of the spatiotemporal LUR modelling performance with different radius buffer of land use data extraction.

Buffer radius of data extraction (m)	Modelling performance	
	R^2^ (%)	RMSE (μg/m^3^)
300	88.54	5.73
500	88.37	5.77
800	88.64	5.70
1000	88.75	5.67
1300	88.86	5.65
1500	88.86	5.65
1800	88.86	5.65
2000	88.71	5.69

*Note*: The buffer radiuses were used for the data extraction of road density (length, meters) and land use data (farmland, blue space, living land, and green space).

R^2^: R square.

RMSE: Root mean squared prediction error (μg/m^3^).

All statistical analyses were performed using R software (version 3.5.1), and the SpatioTemporal (version 1.1.9) *R* packages were mainly used.

### Interpretation

The ST-LUR model using meteorology and GIS covariates could well predict the spatial and temporal variability of ambient PM_2.5_, and precisely assess individual level long-term exposure. This method has the potential to link with a wide range of health data and help understand health impacts of air pollution.

## Declaration of Competing Interest

The authors declare no conflict of interest.
